# An improved algorithm for rapid diagnosis of pleural tuberculosis from pleural effusion by combined testing with GeneXpert MTB/RIF and an anti-LAM antibody-based assay

**DOI:** 10.1186/s12879-019-4166-1

**Published:** 2019-06-21

**Authors:** Qingtao Liang, Yu Pang, Yang Yang, Hua Li, Chao Guo, Xinting Yang, Xiaoyou Chen

**Affiliations:** 1 Department of Tuberculosis, Beijing Chest Hospital, Capital Medical University, Beijing Tuberculosis and Thoracic Tumor Research Institute, Beijing, China; 2 National Clinical Laboratory on Tuberculosis, Beijing Key Laboratory on Drug-Resistant Tuberculosis Research, Beijing Chest Hospital, Capital Medical University, Beijing Tuberculosis and Thoracic Tumor Research Institute, Beijing, China

**Keywords:** Tuberculosis, Pleural, Diagnose, Lipoarabinomannan

## Abstract

**Background:**

This retrospective study evaluated the performance of a lipoarabinomannan (LAM)-based immunological method for diagnosing pleural tuberculosis (TB) from pleural effusion samples. Results were compared to those obtained using conventional culture and molecular testing methods.

**Methods:**

Suspected pleural TB patients who visited Beijing Chest Hospital for medical care between January 2016 and June 2017 were retrospectively analysed in the study. Pleural effusion samples were tested for *Mycobacterium tuberculosis* (MTB) using the BACTEC MGIT 960 System, GeneXpert, and an anti-LAM antibody assay (LAM assay).

**Results:**

Pleural effusion samples were collected from a total of 219 retrospectively recruited participants suspected of having pleural TB. Thirteen of 155 confirmed pleural TB cases tested positive for MTB via MGIT culture, for a sensitivity of 8.4% [95% confidence interval (CI): 4.0–12.8%]. In addition, GeneXpert and LAM testing identified 22 and 55 pleural TB cases, for sensitivities of 14.2% (95% CI: 8.7–19.7%) and 35.5% (95% CI: 28.1–43.6%), respectively. The specificities of these two assays were 100.0% (95% CI: 92.9–100.0%) and 96.9% (95% CI: 88.2–99.5%), respectively. Combined application of culture and LAM testing identified 60 positive cases, for a sensitivity of 38.7% (95% CI: 31.0–46.4%) that was significantly higher than that of MGIT culture alone (*P* < 0.01). Similarly, use of LAM testing in combination with GeneXpert led to correct diagnosis of 40.0% (95% CI: 32.3–47.7%) of pleural TB cases, a higher rate than obtained using GeneXpert alone (*P* < 0.01). In addition, the specificity of the combined assay of GeneXpert and LAM testing was 96.9% (95% CI: 88.2–99.5%). Patients aged 25 to 44 years were more likely to have positive LAM assay results than those ≥65 years of age (*P* = 0.02). Meanwhile, the proportion of diabetic patients with positive LAM assay results was significantly lower than that of the non-diabetes group (*P* = 0.03).

**Conclusions:**

An anti-LAM antibody detection assay showed potential for diagnosis of pleural TB from pleural effusion samples. Combined use of the LAM assay with MGIT culture or GeneXpert methods could improve sensitivity for improved pleural TB diagnosis compared to results of individual conventional tests alone.

## Background

Tuberculosis (TB), caused by *Mycobacterium tuberculosis* complex (MTBC or MTB), is a serious global public health concern [1]. Despite great progress made in recent decades toward reducing TB disease burden, 10.1 million incident cases and 1.6 million deaths are currently observed each year worldwide [1]. In addition to damaging the lungs as the most commonly affected tissue, tuberculosis can involve any other organ or tissue to cause so-called extrapulmonary tuberculosis (EPTB) [2]. Globally, reported proportions of extrapulmonary cases range from 15 to 25% across countries [2, 3], with a worsening burden of EPTB disease resulting from patient co-infection with human immunodeficiency virus (HIV) [2]. Unfortunately, this TB/HIV co-infection scenario has been relatively neglected by TB control programs, mainly due to its low overall contribution to TB transmission within the community [4]. Moreover, although pulmonary TB cases are often easily recognizable due to typical radiological features and positive bacteriological evidence, EPTB is frequently more difficult to diagnose due to non-specific clinical and radiological features of EPTB that are often subject to variable interpretation [5].

Tuberculous pleurisy, one of the most common manifestations of EPTB, is the most common cause of pleural effusion in patients in many countries [6, 7]. As for other forms of EPTB, pleural TB poses a great diagnostic challenge, since conventional acid-fast bacilli (AFB) and culture methods have poor sensitivity as tools for diagnosing this disease [8]. A promising approach, PCR, has been applied to the detection of mycobacterial DNA in pleural fluid, with molecular diagnostic sensitivity ranging from 29 to 75% depending on target sequence amplified and DNA extraction procedure [9–11]. Recently, the use of GeneXpert, a fully automatic molecular diagnostic system, provides rapid and accurate detection of EPTB. However, this assay can be difficult to perform in resource-limited countries, due to high cartridge costs and infrastructural requirements [12]. Additionally, given the poor sensitivity of GeneXpert in pleural TB, WHO has not recommended the use of GeneXpert for the diagnosis of pleural TB [13–15]. Therefore, the lack of a reliable test for detecting MTB in pleural effusion specimens undoubtedly leads to misdiagnosis or missed diagnosis of pleural TB. These challenges thus highlight the urgent need for development of more effective diagnostics for timely diagnosis of pleural TB [12].

Pleural TB results from entry of MTB antigens into the pleural space after the rupture of a subpleural focus. This entry of antigens leads to generation of a host antibody response and accumulation of pleural effusion that result from a hypersensitivity reaction [16]. Therefore, immunological tests based on detection of antibodies present in pleural effusion specimens offer promise for improving pleural TB diagnosis [17]. Performance of immunologically based testing had been previously evaluated as a tool for diagnosing pleural TB from blood samples [17], with sensitivity estimates ranging from 26 to 59% and specificity estimates ranging from 81 to 100% [2, 18, 19]. Despite the fact that the WHO has advised against the use of commercial serological tests for diagnosing active TB [20], a serological test for diagnosis of pleural TB may be of value, since current tools are not diagnostically effective for this disease. Such a test would be based on detection of anti-MTB antibodies produced by the underlying inflammatory reaction that leads to pleural TB. This concept is based on limited data from previous studies, which had demonstrated that lipoarabinomannan (LAM), an integral component of the MTB cell wall, can stimulate production of anti-LAM antibodies by the human host [21]. We therefore conducted a retrospective study to assess the performance of an immunological method (the LAM assay) to detect anti-LAM antibodies in TB pleural effusion specimens. We then compared LAM assay sensitivity and specificity to corresponding results obtained using conventional culture and molecular testing methods.

## Methods

### Study subjects

We retrospectively analyzed patients suspected of having pleural TB who visited Beijing Chest Hospital to seek medical care between January 2016 and June 2017. Inclusion criteria were: i) suspected pleural TB based on standard clinical and radiological criteria, including a persistent cough of 2 weeks or more, unexplained fever for 2 weeks or more, weight loss, and radiological evidence of pleural effusion; and ii) results of patient pleural effusion specimen testing performed via mycobacterial culture, GeneXpert and anti-LAM antibody test (LAM assay) methods. Patients who were receiving anti-TB treatment were excluded from the final analysis. Participant demographic profiles and clinical information were obtained from medical records.

**Fig. 1:**
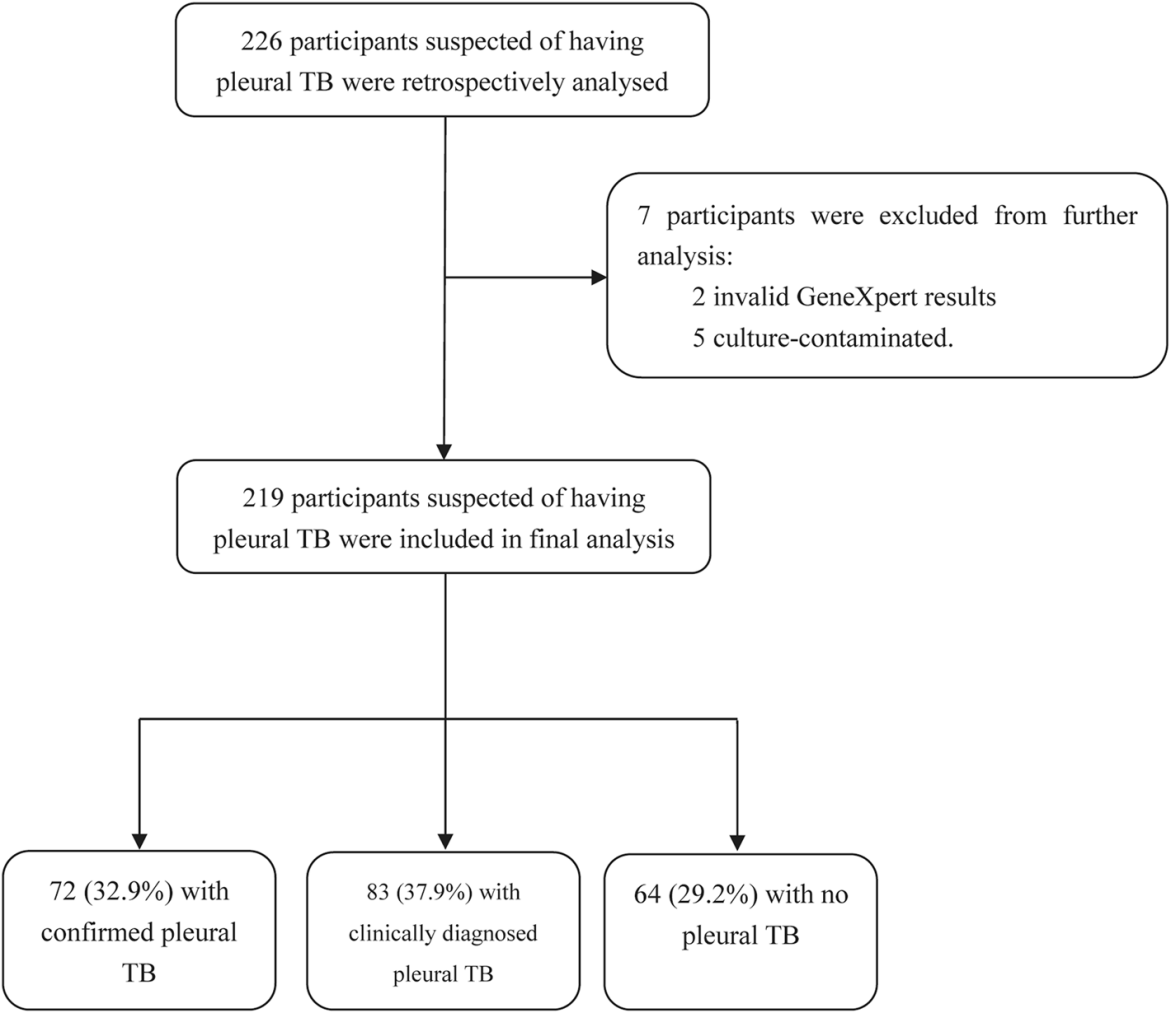
Classification of patients with suspect pleural tuberculosis

A total of 226 participants suspected of having pleural TB were retrospectively reviewed. Of the 226 cases, 7 were excluded due to invalid GeneXpert results (*n* = 2) and contaminated culture results (*n* = 5). Ultimately, results from 219 patients were used in the final analysis. On the basis of laboratory examination results and clinical symptoms, 72 cases (32.9%) belonged to confirmed pleural TB, 83 (37.9%) to clinically diagnosed pleural TB, and 64 (29.2%) to non-pleural TB. Forty-seven (73.4%, 47/64) non-pleural TB cases were afflicted with malignancies and the other 17 cases (26.6%, 17/64) with pneumonia (Fig. 1).

### Diagnostic criteria

A combination of clinical, microbiological, histological, and radiological findings was used for pleural TB diagnostic confirmation according to Chinese national guidelines [22]. Briefly, pleural effusion specimens obtained from patients suspected of having pleural TB were subjected to routine laboratory analysis, including AFB smear, mycobacterial culture, GeneXpert, and other tests. Pleural TB cases were classified as confirmed pleural TB cases and clinically diagnosed pleural TB cases based on laboratory examination results and clinical symptoms, respectively. Patients with at least one positive MTB culture result via conventional culture method or GeneXpert and/or granulomatous inflammation suggestive of TB from histological examination of pleural biopsy tissue samples were defined as confirmed pleural TB cases. Patients without any experimental diagnostic evidence, but who had exhibited clinical-radiological manifestations based on clinical symptoms prior to receiving treatment for TB, were defined as clinically diagnosed pleural TB cases [11].

### Laboratory examination

Pleural effusion samples were collected in sterile 4-ml tubes and transported to the laboratory for examination. A volume of 2.0 mL of each pleural effusion sample was centrifugated at 3000×g for 15 min then each pellet was resuspended in phosphate buffered saline (PBS). A volume of 500 mL of each suspension was inoculated into a separate 7-mL MGIT tube supplemented with 0.8 mL of oleic acid-albumin-dextrose-catalase (OADC) along with PANTA™. MGIT tubes were placed into the MGIT 960 instrument and cultures with growth were automatically reported by the instrument. Species identification testing was performed for all positive cultures using the Tibilia Rapid Test (Chuangxin, Hangzhou, China) [23].

For GeneXpert MTB/RIF testing, 1.0 mL of pleural effusion was mixed with 2.0 mL of sample reagent followed by incubation at room temperature for 15 min. 2.0 mL of each inactivated sample mixture was transferred to a separate Xpert MTB/RIF cartridge then cartridges were inserted into the GeneXpert instrument. Results confirming the presence of MTB were automatically reported by the instrument within 90 min [24].

Detection of anti-LAM antibodies was performed according to the manufacturer’s instructions (Biovision, Beijing, China). Briefly, a volume of 10 μL of pleural effusion was tested for the presence of antibodies specific for highly purified LAM antigen immobilized to test strips. LAM antigen had been secreted by MTB during active infection prior to purification. When a pleural effusion sample was applied to a test strip, it contacted the antigen line on the strip to permit antibodies in the specimen to bind to antigen on the strip. Strips were incubated for 10 min at room temperature. Bound antibody was detected using goat anti-human IgG antibody conjugated to horseradish peroxidase. Antigen-antibody complexes were demonstrated by the presence of a pink line formed by antibody-conjugated horseradish peroxidase (within immunocomplexes) that reacted with the substrate solution to produce a colored line. A line with intensity equal or greater than that of the positive control was recorded as a positive result.

### Statistical analysis

In view of the low overall rate of positive results obtained for pleural TB patients, a composite reference standard (CRS) was created from clinically diagnosed pleural TB case samples and from cases with laboratory confirmation for use as the gold standard. Results obtained from testing the CRS using different methods and combinations of methods were compared. Samples from patients with malignancies and other respiratory diseases served as negative controls. Sensitivity, specificity, positive predictive value (PPV), and negative predictive value were calculated to evaluate the performance of diagnostic tests for detection of pleural TB. The chi-square test was used to compare categorical variables and differences were declared significant for *P* < 0.05. All statistical analyses were performed using SPSS version 17.0 (Chicago, IL, USA).

## Results

### Participants

We first compared the distribution of demographic characteristics between pleural and non-pleural TB cases. As summarized in Table 1, we observed that the percentage of male patients in the pleural TB group was significantly higher than that of female patients (odds ratio (OR) [95% confidence interval (CI)]: 4.66[1.81–11.97], *P* < 0.01). In addition, percentages of pleural patients aged < 25 years (OR [95% CI]: 4.00[1.03–15.60], *P* = 0.04) and 25–44 years (OR [95% CI]: 2.82[1.14–6.98], *P* = 0.02) were significantly higher than in the non-pleural TB group. By contrast, no significant difference was observed with regard to residence or diabetes status between pleural TB and non-pleural TB groups (*P* > 0.05).

**Table 1:** Demographic characteristics of participants suspected of having pleura TB

Characteristics	Diagnostic Class				
	Pleural TB cases (155) *n* (%)	Non pleural TB cases (64) *n* (%)	Odds ratios (95% CI)	*P* value	Total (219) *n* (%)
Sex					
Male	121 (78.1)	37 (57.4)	2.60 (1.39–4.85)	< 0.01	158 (72.1)
Female	34 (21.9)	27 (42.6)	1.00	Ref.	61 (27.9)
Age group (years)					
< 25	24 (15.5)	3 (4.9)	4.00 (1.03–15.60)	0.04	27 (12.3)
25~44	62 (40.0)	11 (18.0)	2.82 (1.14–6.98)	0.02	73 (33.3)
45~64	41 (26.5)	36 (59.0)	0.57 (0.26–1.24)	0.16	77 (35.2)
≥ 65	28 (18.1)	14 (23.0)	1.00	Ref.	42 (19.2)
Residence					
Rural	77 (57.4)	29 (63.9)	1.19 (0.66–2.14)	0.56	106 (48.4)
Urban	78 (42.6)	35 (41.0)	1.00	Ref.	113 (51.6)
Diabetes					
Yes	17 (11.0)	7 (11.5)	1.00 (0.40–2.55)	0.96	24 (11.0)
No	138 (89.0)	57 (88.5)	1.00	Ref.	195 (89.0)

**Table 2:** Performance of diagnostics for the diagnosis of pleural tuberculosis in pleural effusion samples

Method	Sensitivity 95% CI	Specificity 95% CI	PPV^a^ 95% CI	NPV 95% CI
Culture	8.4% (13/155)	100.0% (64/64)	100.0% (13/13)	31.1% (64/206)
	(4.0–12.8%)	(92.9–100.0%)	(100.0–100.0%)	(24.7–37.4%)
GeneXpert	14.2% (22/155)	100.0% (64/64)	100.0% (22/22)	32.5% (64/197)
	(8.7–19.7%)	(92.9–100.0%)	(100.0–100.0%)	(25.9–39.0%)
LAM	35.5% (55/155)	96.9% (62/64)	96.5% (55/57)	38.3% (62/162)
	(28.1–43.6%)	(88.2–99.5%)	(91.7–100.0%)	(30.8–45.8%)
Culture+GeneXpert	14.8% (23/155)	100.0% (64/64)	100.0% (23/23)	32.7% (64/196)
	(9.2–20.4%)	(92.9–100.0%)	(100.0–100.0%)	(26.1–39.2%)
Culture+LAM	38.7% (60/155)	96.9% (62/64)	96.8% (60/62)	39.5% (62/157)
	(31.0–46.4%)	(88.2–99.5%)	(92.4–100.0%)	(31.8–47.1%)
GeneXpert+LAM	40.0% (62/155)	96.9% (62/64)	96.9% (62/64)	40.0% (62/155)
	(32.3–47.7%)	(88.2–99.5%)	(92.6–100.0%)	(32.3–47.7%)
Culture+GeneXpert+LAM	40.6% (63/155)	96.9% (62/64)	96.9% (63/65)	40.3% (62/154)
	(32.9–48.4%)	(88.2–99.5%)	(92.7–100.0%)	(32.5–48.0%)

^a^*PPV* positive predictive value, *NPV* negative predictive value

^b^*χ*^2^ = 33.23, *P* < 0.01(Culture Se. vs. LAM Se.); *χ*^2^ = 18.82, *P* < 0.01(GeneXpert Se.vs. LAM Se.); *χ*^2^ = 2.61, *P* = 0.11(Culture Se.vs. GeneXpert Se.); *χ*^2^ = 39.58, *P* < 0.01(Culture Se. vs. Culture+LAM Se.); *χ*^2^ = 26.13, *P* < 0.01(GeneXpert Se.vs. GeneXpert+LAM Se.); *χ*^2^ = 3.14, *P* = 0.08(Culture Se.vs. Culture+GeneXpert Se)

### Performance of laboratory diagnostics

Thirteen of 155 pleural TB cases were detected by MGIT culture testing, for a sensitivity of 8.4% (95% CI: 4.0–12.8%). In addition, GeneXpert and LAM testing identified 22 and 55 pleural TB cases, for a sensitivity of 14.2% (95% CI: 8.7–19.7%) and 35.5% (95% CI: 28.1–43.6%), respectively. LAM assay sensitivity was significantly higher than that of MGIT culture and GeneXpert (*P* < 0.01). No significant difference was observed in sensitivity between MGIT culture and GeneXpert methods for pleural TB detection from pleural effusion samples (*P* > 0.05).

We further analyzed the performance of combined diagnostic testing for pleural TB diagnosis. When combining MGIT culture and GeneXpert, one additional positive patient was detected, yielding a sensitivity of 14.8% (95% CI: 9.2–20.4%) and specificity of 96.9% (95% CI: 92.9–100.0%), while the difference between MGIT culture and MGIT culture+GeneXpert was not statistically significant (*P* = 0.08). In contrast, the combined application of culture-based testing and LAM assay identified 60 positive cases, resulting in a sensitivity of 38.7% (95% CI: 31.0–46.4%), which was significantly higher than that of MGIT culture alone (*P* < 0.01). Similarly, by using the LAM assay in combination with GeneXpert, 40.0% (62/155, 95% CI: 46.0–62.2%) of pleural TB cases were correctly diagnosed, a higher detection rate than that obtained using GeneXpert alone (*P* < 0.01) (Table 2).

### Factors associated with negative LAM results

We compared distributions of demographic and clinical characteristics between LAM-positive and LAM-negative groups (Table 3). Compared with the percentage of LAM-positive patients in the ≥65 years age group (9.1%), the percentage of LAM-positive patients in the 25–44 years group was higher (OR [95% CI]: 3.55[1.19–10.55], *P* = 0.02), with no statistical difference observed for the group of patients aged < 25 years (OR [95% CI]: 3.29[0.93–11.61], *P* = 0.06) or the group of patients aged 45–64 years (OR [95% CI]: 2.22[0.69–7.15], *P* = 0.18). In addition, the proportion of patients in the diabetes group with positive LAM assay results (5.5%) was significantly lower than that of the non-diabetes group (14.0%, OR [95% CI]: 0.22[0.05–0.99], *P* = 0.03).

**Table 3:** Factors associated with LAM-based results among pleural TB patients

Characteristics	LAM positive (*n* = 55)		LAM negative (*n* = 100)		Odds ratios (95% CI)	*P* value
	No.	Col %	No.	Col %		
Sex						
Male	44	80.0	77	77.0	1.20(0.53–2.68)	0.67
Female	11	20.0	23	23.0	1.00	Ref.
Age group (years)						
< 25	10	18.2	14	14.0	3.29 (0.93–11.61)	0.06
25~44	27	49.1	35	35.0	3.55 (1.19–10.55)	0.02
45~64	13	23.6	27	27.0	2.22 (0.69–7.15)	0.18
≥ 65	5	9.1	23	23.0	1.00	Ref.
Residence						
Rural	26	47.3	51	51.0	0.86 (0.45–1.67)	0.66
Urban	29	52.7	49	49.0	1.00	Ref.
Diabetes						
Yes	2	5.5	15	14.0	0.22(0.05–0.99)	0.03
No	52	94.5	86	86.0	1.00	Ref.

## Discussion

The diagnosis of pleural TB is still an unsolved problem worldwide due to unreliable laboratory detection test results [12]. Numerous studies have documented that the GeneXpert assay is a useful confirmatory (rule in) diagnostic test for EPTB from various types of clinical specimens, such as cerebrospinal fluid and tissue samples [25–27]. However, it is not endorsed for use as an initial test for diagnosing patients suspected of having pleural TB, due to its unsatisfactory performance for testing of pleural effusion specimens [28, 29]. In this study, detection of anti-LAM antibody in pleural effusion samples showed high specificity and moderate sensitivity for diagnosis of pleural TB, as compared with GeneXpert and MGIT culture.

Here, the LAM assay could identify approximately 40% of pleural TB cases when combined with MGIT culture or GeneXpert, a success rate significantly higher than obtained by each test alone. However, the combination of MGIT culture and GeneXpert offered limited benefit for diagnosing pleural TB from pleural effusion samples, since positive results of these etiological diagnostic methods are based on the presence of tubercle bacilli in specimens. Moreover, despite different detection limits between culture and GeneXpert methods, a high proportion of detection overlap between the two methods would undoubtedly weaken justification for their combined use for pleural TB diagnosis. On the contrary, detection of anti-LAM antibodies yielded better positivity despite the fact there were few tubercle bacilli but there were adequate LAM molecules in circulation in bodily fluids of TB patients and that could elicit significant production of anti-LAM antibodies and thus gave reasonably good sensitivity.

Notably, numerous studies have demonstrated that EPTB has been reported more frequently in females than in males [3, 30]. Conversely, in this report we found that men were more likely to have pleural TB. However, a nationwide epidemiological study from the Netherlands could reconcile these conflicting results, since in that study EPTB was relatively more prevalent among females, while pleural TB was more common in males (8.6% in males versus 6.7% in females, *P* < 0.01) [5]. Although the exact causes for gender biases among TB-related diseases remain unknown, we hypothesize that immunity, hormones, and socio-economic factors may be involved [31], warranting further research. Meanwhile, our analysis showed that pleural TB was more frequently observed among patients younger than 44 years of age. One possible explanation may be due to the fact that pleural TB is likely a manifestation of paucibacillary mycobacterial infection that leads to an immunologically-based hypersensitivity response [16]. Therefore, a decline in immunity with increasing age may decrease the risk of pleural TB occurrence in aging TB patients [32]. In line with this hypothesis, further analysis here revealed that younger pleural TB patients were more likely to generate an anti-LAM antibody response than were elderly patients.

In addition to advancing age, diabetes is another major comorbidity associated with low rates of pleural TB diagnosis that may contribute to false-negative LAM assay results. It is now well accepted that both humoral and cellular immunity are involved in the pathogenesis of diabetes [33], since patients with diabetes appear to exhibit reduced humoral immunity, with more rapid decay of antibody responses than observed in non-diabetic patients [33]. Therefore, poorer humoral immune function of patients in this population may have led to a lower pleural TB detection rate using the anti-LAM antibody detection method. However, it should be noted that the small number of diabetic patients in our study population may have undermined the reliability of these results. Nevertheless, the low detection rate obtained using the LAM assay highlights the need for new specific biomarkers that are more suitable for detecting pleural TB from pleural effusion samples in patients with diabetes.

Compared with other diagnostic tests for pleural TB, the LAM assay is simple and easy to perform and generates results within 1 h after sample collection. Moreover, the cost of this assay is less than 1.00 USD per test, which justifies its use as a cost-effective test for detecting pleural TB. Furthermore, since special equipment and skilled operators are not always provided to TB laboratories in resource-limited settings, another advantage of this assay is that it can be operated and interpreted without any complicated procedures or sophisticated instrumentation. Therefore, the LAM assay is an affordable and promising diagnostic test for use in diagnosis of pleural TB, especially in resource-limited settings.

This study had several obvious limitations. First, this was a retrospective study rather than one based on continuous recruitment of individuals with suspected pleural TB, which may limit the overall significance of our study conclusions. Second, the positive rate of MGIT culture for detection of MTB in pleural effusion specimens obtained here was lower than rates obtained in previous studies, a result possibly due to the small sample volume used for mycobacterial culture. As a consequence, the relatively low sensitivity may have negated the potentially beneficial role of mycobacterial culture for analysis of various diagnostic combinations. Third, we realize that the performance of the LAM assay is far from acceptable for applications needed for high-priority target product profiles [34]. The combination use of multiple biomarkers, such as 38kD, 16kD, Ag85A and MPT64 [35], may improve the sensitivity of this assay for analysis of pleural TB using pleural effusion specimens. Fourth, the performance of the LAM assay was only evaluated in pleural effusion samples rather than serum samples in this study. Despite these limitations, our study provides important insights into the use of an anti-LAM antibody-based assay for diagnosis of pleural TB.

## Conclusions

The results of this study demonstrate that the LAM assay, which detects patient anti-LAM antibodies, shows promising performance for achieving pleural TB diagnosis from pleural effusion samples. Moreover, the combined use of the LAM assay with MGIT culture or GeneXpert could improve pleural TB diagnostic sensitivity relative to each test alone. In addition, old age and diabetes comorbidities are main factors associated with false-negative LAM assay results.

## Data Availability

The datasets used and/or analysed during the current study available from the corresponding author on reasonable request.

## Abbreviations

AFB: Acid-fast bacilli
EPTB: Extrapulmonary tuberculosis
HIV: Human immunodeficiency virus
LAM: Lipoarabinomannan
MTBC: *Mycobacterium tuberculosis* complex
OADC: Oleic acid-albumin-dextrose-catalase
TB: Tuberculosis
